# Defining Acute Traumatic Encephalopathy: Methods of the “HEAD Injury Serum Markers and Multi-Modalities for Assessing Response to Trauma” (HeadSMART II) Study

**DOI:** 10.3389/fneur.2021.733712

**Published:** 2021-12-08

**Authors:** W. Frank Peacock, Damon Kuehl, Jeff Bazarian, Adam J. Singer, Chad Cannon, Zubaid Rafique, James P. d'Etienne, Robert Welch, Carol Clark, Ramon Diaz-Arrastia

**Affiliations:** ^1^Baylor College of Medicine, Houston, TX, United States; ^2^Carillion Clinic, Roanoke, VA, United States; ^3^Department of Emergency Medicine, University of Rochester, Rochester, NY, United States; ^4^Renaissance School of Medicine at Stony Brook University, Stony Brook, NY, United States; ^5^University of Kansas Medical Center, Kansas City, MO, United States; ^6^Integrative Emergency Service/John Peter Smith Health System, Fort Worth, TX, United States; ^7^Detroit Medical Center, Detroit, MI, United States; ^8^William Beaumont Hospital, Royal Oak, MI, United States; ^9^Department of Emergency Medicine, University of Pennsylvania, Philadelphia, PA, United States

**Keywords:** traumatic brain injury, neuropsychiatric testing for TBI, biomarkers for TBI, diagnosis of TBI, prognosis of TBI

## Abstract

Despite an estimated 2.8 million annual ED visits, traumatic brain injury (TBI) is a syndromic diagnosis largely based on report of loss of consciousness, post-traumatic amnesia, and/or confusion, without readily available objective diagnostic tests at the time of presentation, nor an ability to identify a patient's prognosis at the time of injury. The recognition that “mild” forms of TBI and even sub-clinical impacts can result in persistent neuropsychiatric consequences, particularly when repetitive, highlights the need for objective assessments that can complement the clinical diagnosis and provide prognostic information about long-term outcomes. Biomarkers and neurocognitive testing can identify brain injured patients and those likely to have post-concussive symptoms, regardless of imaging testing results, thus providing a physiologic basis for a diagnosis of acute traumatic encephalopathy (ATE). The goal of the HeadSMART II (HEAD injury Serum markers and Multi-modalities for Assessing Response to Trauma) clinical study is to develop an *in-vitro* diagnostic test for ATE. The BRAINBox TBI Test will be developed in the current clinical study to serve as an aid in evaluation of patients with ATE by incorporating blood protein biomarkers, clinical assessments, and tools to measure, identify, and define associated pathologic evidence and neurocognitive impairments. This protocol proposes to collect data on TBI subjects by a multi-modality approach that includes serum biomarkers, clinical assessments, neurocognitive performance, and neuropsychological characteristics, to determine the accuracy of the BRAINBox TBI test as an aid to the diagnosis of ATE, defined herein, and to objectively determine a patient's risk of developing post-concussive symptoms.

## Background

Mild traumatic brain injury (mTBI) annually affects over 23 million people worldwide (1). In 2014, there were an estimated 2.5 million TBI-related emergency department (ED) visits in the US, of which 90% were estimated to be the result of mTBI (2, 3). From 2006 to 2014, the number of TBI-related ED visits increased by 53% (4), although the real incidence is likely much higher, as mTBI is underdiagnosed in the ED (5, 6), patients may present to alternate venues (e.g., primary care), or they do not seek care at all. The majority of adults over age 40 report a history of head injury with loss of consciousness at some point in their lives (4, 7–9).

Over 90% of TBI is classified as “mild” TBI, a term that has been criticized as misleading, since many of these injuries result in significant disabilities (10), especially if they are repetitive (11). Despite a classification of “mild,” TBI can cause persistent cognitive and physical symptoms with major impacts on affected patients function and quality of life (12, 13). In 2010 an estimated 2% of the U.S. population lived with TBI-related disabilities, at an annual estimated expense of about $77 billion (4).

Despite the magnitude of this global public health problem, the diagnosis and classification of mTBI remains challenging. The terms mTBI and concussion (which has no accepted standard definition) continue to be used interchangeably, with a 2014 review reporting 50 different mTBI definitions across 101 studies (14). Although the NINDS have developed TBI common data elements (15), the lack of objective diagnostic or prognostic tools for mTBI severely limits its effectiveness.

To date, few U.S. Food and Drug Administration (FDA) cleared strategies are available to objectively diagnose, or define prognosis, in patients presenting with head injury. While anatomic imaging methods, generally with computerized tomography (CT), show TBI-related abnormalities in ~10% of patients evaluated in EDs, a significant number of patients have disabling TBI symptoms despite initial negative imaging. When neuroimaging is negative, there are few currently available objective tests to identify significant occult injury and predict future dysfunction. Consequently, while acute care physicians can exclude emergencies that require neurosurgical intervention, or neurocritical care (e.g., intracranial hemorrhage) by imaging or physical examination, and they can perform testing to exclude the need for imaging (16), they have limited ability to objectively diagnose mTBI or identify which patients will suffer longer term poor outcomes. In fact, one ED study demonstrated that the ability of physicians to prospectively identify patients who will have subsequent mTBI related symptoms 90 days after their presentation is extremely poor, with a sensitivity of only 8.1% and a specificity of 54.5% (17).

Diagnosis of mTBI in the ED is currently based on clinical findings, which can be problematic, since the medical history is often incomplete, symptoms are vague, and the physical signs are non-specific. These challenges are further confounded by factors such as intoxication or pre-existing neurologic impairments (18). The recent availability of biomarker testing may allow for more precise identification of patients with mTBI, as a manifestation of acute traumatic encephalopathy (ATE), by adding the objective evidence of injury-related leakage of brain-derived proteins into the blood (16). Although ATE testing is currently unavailable in the acute care environment, the ATE cohort is identified as having abnormal biomarkers and/or neurocognitive dysfunction, irrespective of the results of neuroimaging. Ultimately, this strategy may be applied to risk stratify patients by determining their likelihood of developing downstream symptoms caused by their TBI.

In addition to biomarkers, evidence also supports the value of adding neurocognitive (NC) assessments for the characterization of functional deficits at the time of injury (19). The use of NC testing to identify patients at risk for protracted or disabling neurological deficits following head injury, irrespective of an imaging evaluation, may allow the selection of patients who will benefit from targeted interventions that have the potential to improve patient outcomes (20–24).

BRAINBox TBI Test is a novel technology that uses a multi-marker serum panel, in conjunction with computerized neurocognitive testing, to aid in an objective diagnosis of ATE. In addition, results of this testing strategy may identify those at risk for of persistent symptoms, i.e., it provides a prognostic determination. Thus, our purpose is to determine the ability of BRAINBox TBI Test technology to identify patients with ATE, as well as to define their prognosis, in real-world patients presenting with suspected ATE.

## Methods

Our primary objectives are to determine the ability of the BRAINBox TBI Test to:

1. Diagnose ATE using statistical modeling methods that combine blood biomarker values, focused patient health information, and NC/NP testing.
2. Predict persistent symptoms up to 90 days after a diagnosis of ATE by using statistical modeling combining biomarkers, focused patient health information, and NC/NP testing.

HeadSMART II (HEAD injury Serum markers and Multi-modalities for Assessing Response to Trauma) is a clinical study that was devised to provide the information for these objectives and to provide clinical validation of the test. This is a multicenter, international, observational study, NCT04423198, with an expectation of enrolling up to 2000 subjects. The study started Q1 2021, with enrollment anticipated to be completed by the end of 2022. Subjects include those with suspected ATE, and control populations. Inclusion and exclusion criteria are provided in Table 1. Data compliant with Common Data Elements; Modular Data Elements for Traumatic Brain Injury (25), as well as ICD codes, will be collected at the index visit (*t* = 0), and at 14-, 30- and 90-days post-injury in suspected ATE patients, at the index visit and at 14 days for trauma control patients, and only at the index visit for healthy control subjects. All participants will provide 2 blood draws (separated by 1–4 h) and NC assessments at enrollment, and with added symptom specific assessments and neuropsychological testing (patient reported outcomes, PROs) at their follow up visits. Blood draws will consist of obtaining serum, plasma (including optional collection for DNA analysis) and PAXGene tubes, which are processed according to manufacturer's recommended protocols, then stored at −80C, and shipped to a core lab for biomarker analysis. Biomarker analysis will use BRAINBox's proprietary serum/plasma biomarker assays including, but not limited to, GFAP (Glial Fibrillary Acidic Protein), NSE (Neuron Specific Enolase-2), NRGN (Neurogranin), SNCB (Beta-Synuclein), and MT3 (Metallothionein-3). These biomarkers were chosen due to their demonstrated diagnostic and prognostic utility for TBI in the HEADSMART pilot trial (26–30).

**Table 1:** Inclusion and exclusion criteria.

**A. Inclusion Criteria: Suspected ATE subjects**
1. Presents with a blunt head trauma
2. Age ≥ 18 years, and ability to provide a blood sample within 96 h of injury
3. Ability to provide informed consent. Consent may be obtained with assistance of a legally authorized representative (LAR)
4. Presents with a blunt head trauma
**B. Exclusion Criteria: Suspected ATE and Trauma Control subjects**
1. Glasgow Coma Scale (GCS) score <13, at time of screening
2. Need for general anesthesia at the time of presentation in the ED
3. Diagnosed dementia requiring assistance for daily living
4. Any head trauma requiring medical attention from a physician within the last 6 months
5. Received chemotherapy or radiation within the last year
6. History of stroke with disabling outcomes, brain tumor, epilepsy or intracranial surgery/hemorrhage
7. Psychiatric hospitalization in the last 90 days
8. Blood transfusion within the prior 4 weeks
9. Non-working telephone number
10. Current participant in an interventional clinical trial
11. Cannot perform study tasks on an iPad (e.g., not wearing corrective lenses necessary to read, inability to use both hands)
12. Subject unsuitable for participation, as determined by their physician, or any research staff
**C. Inclusions for Trauma and Healthy Controls**
1. Age ≥ 18 years
2. Ability to provide a blood sample; (For Trauma Controls within 96 h of injury)
3. Ability to provide informed consent. (For Trauma Controls consent may be obtained with assistance of a legally authorized representative)
4. Presents with at least one injury requiring an X-Ray (TC's only)
**D. Exclusions for Healthy Controls (HC's)**
1. Head trauma or symptoms with head trauma at presentation
2. Head trauma requiring medical attention from a physician within the last 6 months
3. Internal organ injury that requires inpatient hospitalization
4. Need for general anesthesia at the time of presentation in the ED
5. Diagnosed dementia requiring assistance for daily living
6. Received chemotherapy or radiation within the last year
7. History of stroke with disabling outcomes, brain tumor, epilepsy or intracranial surgery/hemorrhage
8. Psychiatric hospitalization in the last 90 days
9. Blood transfusion within the prior 4 weeks
10. Non-working telephone number or participant in an interventional clinical trial
12. Cannot perform study tasks on an iPad (e.g., no glasses necessary to read)
13. Subject considered unsuitable for participation by physician, or any research staff

The blood biomarker values are quantified by comparing the raw signal detected from replicates to a calibrant curve that covers a concentration range greater than the clinical range of the samples tested. The replicates are evaluated for specifications including coefficient of variation that determine the technical validity of the test. All samples passing acceptance criteria are reported as a quantity (amount per unit volume, e.g., picograms/milliliter). The biomarker values will be used as predictors in the statistical/machine learning algorithms.

The BrainCheck Application will be used to provide digitized neurocognitive assessments at each visit, the details of which are given in Table 2. BrainCheck uses digitized versions of several previously validated neurocognitive pen and paper tests, each with associated metrics of function such as duration and accuracy of responses. These comprise a composite battery and that also includes measures of balance and coordination, functions that can be affected by acute brain injury. For each subject, the data are compared against a normative/reference database previously developed by BrainCheck. The normalized values will also be used as predictors in the statistical/machine learning algorithms.

**Table 2:** Neuropsychiatric/neurocognitive testing (19).

**Tests for ATE Score and Prognosis Report**
a. Rivermead (collected on BrainCheck iPad)
b. BrainCheck Cognitive Assessments
i. Flanker Test
ii. Trail Making Test A and B
iii. Digit Symbol Substitution Test
iv. Stroop Test
v. Recall Test
vi. Coordination/Balance Test
**Additional Neurological Tests for use in Prognosis Report**
a. Headache Impact Test (HIT-6)
b. Dizziness Handicap Inventory
c. Convergence Insufficiency Symptom Survey
d. Balance Error Scoring System (BESS)

The clinical data are collected through electronic CRFs and stored in REDCap Cloud Electronic Data Capture software. The NINDS common data elements for general reporting and for traumatic brain injury have been used to guide the design of the study and the CRFs.

Subjects participate in the study for up to 90 days, must enroll within 96 h of blunt head trauma, and have a Glasgow Coma Scale ≥13 at presentation. Up to 1,600 suspected ATE subjects will be enrolled through the study time points to provide sufficient subjects for separate algorithm training and validation cohorts. The demographic breakdown of the subjects will meet good clinical practice guidelines (31). For the determination of prognosis, subjects are first adjudicated as positive for TBI by a diagnostic adjudication committee, who provide an expert clinical diagnosis after reviewing the complete patient history and assessment up to 1-month post-injury. Diagnosed TBI subjects will be further categorized by the presence of symptoms at each study time point.

The Diagnostic Adjudication Committee (DAC) will consist of a panel of physicians experienced in the diagnosis and management of TBI. Two adjudicators, blinded to BRAINBox TBI Test results, will be randomly selected to review each subject's de-identified medical records, physical examination notes, neurological assessments (see Table 2), and core lab neuroimaging report, to define an expert clinical diagnosis of “TBI” or “no TBI.” The adjudication process will begin with each group after the participants have completed their respective follow up visit(s). The expert clinical diagnosis is obtained when 2 panel member's independent diagnoses agree. In the case of diagnostic disagreement, a third panel member performs an independent record review and serves as the tie breaker.

For participants who receive a head CT or MRI as part of standard care, their images are recorded and reviewed by a core lab. The neuroimaging core lab consists of a panel of board-certified neuro-radiologists, two of whom read all images and provide a report to the diagnostic adjudication committee. If the 2 readers do not agree, a 3rd will serve as a tie breaker to define the final report.

Control subjects will be enrolled for the purposes of assay development, including establishing the biomarker reference intervals, and will be cohorted into groups based on age and sex. These will include 120 trauma controls through 14 day follow up, and 120 healthy controls with a single visit. Trauma controls are defined as patients presenting to the ED for a traumatic injury and requiring an X-ray, but who do not have head trauma. Healthy controls will be subjects that report to be healthy and are not taking any prescription medications.

The BRAINBox TBI Test results determining a diagnosis of ATE, will be compared to the expert clinical diagnosis. Adjudicated patients assigned a diagnosis of TBI will be stratified for having high or low risk for post-concussive symptoms in each symptom category, based on biomarker and neuropsychiatric testing, and are defined as either “ATE with” or “ATE without” post-concussive symptoms. The high or low risk for post-concussive symptoms in each symptom category is defined using the validated interpretation guides for each test (Table 3), relevant literature with input from Key Opinion Leaders.

**Table 3:** Symptom categories with corresponding neurocognitive and neuropsychological tests.

**Neuropsychological/ Neurocognitive Test**	**Symptom Category**
Headache Impact Test (HIT-6)	Headache
Dizziness Handicap Inventory, Convergence Insufficiency Symptom Survey, Balance Error Scoring System (BESS)	Motor Impairment (Balance, Dizziness/Vertigo, Visual Dysfunction)
Patient Recorded Outcome Measurement Information System (PROMIS), Sleep Disturbance Short Form	Sleep Disturbance
Glasgow Outcome Scale-Extended (GOSE-E), BrainCheck	Cognitive (Memory, Attention, Concentration, Executive Function)
Generalized Anxiety Disorder (GAD-7), Post-traumatic Stress Disorder Checklist for DSM-5 (PCL-5), Patient Health Questionairre-9 (PHQ-9), and Perceived Stress Scale	Psychological (Depression, Anxiety, Mood, Irritability, Post-traumatic Stress Disorder PTSD)

### Algorithm Training and Testing

Data collected in this study will be used to finalize development of the BRAINBox TBI Test algorithms and to provide clinical validation of test performance. Using subjects in the training cohort, the BRAINBox diagnosis will be made by including the biomarker values, NC assessments and baseline demographics (age and sex) at the initial visit as predictors in a statistical/machine learning algorithm that estimates the ATE score. The decision threshold for ATE will be determined by identifying the ATE score that meets the diagnostic characteristic of a minimum sensitivity and specificity of 85 and 75%, respectively. To create the distributions for “ATE” and “no ATE” groups, the mean and the standard deviation of the score will be calculated on a log odds scale. For those subjects in the validation set, the final algorithm will be applied to the individual's biomarker values, neurocognitive assessments, and baseline demographics (age and sex) at the initial visit. The ATE score will be the output. If the ATE score exceeds the identified threshold, the subject will be classified as having “ATE,” otherwise, the subject will be classified as “no ATE.” A sample's log odds value will be compared to the log odds distribution of the “no ATE” subjects in the training set and a corresponding percentile will be assigned to the sample as the percentile relative to that distribution.

The performance of a prognostic ATE test will be determined by evaluating the biomarker values, neurocognitive assessments, and baseline demographics at the index visit, as predictors of 3 separate models that assign high or low risk to a subject for subsequent symptom occurrence at 14, 30, and 90 days. Symptom categories and tests performed during the study are provided in Table 3. Symptoms are evaluated as a composite at each time point, with thresholds to stratify subjects defined based on the scoring system/scale for each assessment, identifying the subject's risk for a specific symptom category at each time point. A separate statistical/machine learning algorithm will be developed for each time point that calculates the risk at the specific time after injury. In the algorithm, the definition of high or low risk for having post-concussive symptoms is defined by the decision threshold that produces the minimum sensitivity and specificity of 85 and 75%, respectively. For the validation process, the finalized algorithms will be applied to the subject's biomarker values, NC assessments, and baseline demographics (age and sex) at the index visit. If the output of the algorithm exceeds the identified decision threshold, the subject will be classified as high risk. Otherwise, the subject will be classified as low risk for post-concussive symptoms at the specified time point. The prognostic report is designed to aid the clinician in determining the subject's risk of having post-injury post-concussive symptoms at 14, 30, and 90 days.

### Statistical Analysis

#### Sample Size Estimation

Sensitivity and specificity are used to determine performance, since both are essential in the characterization of diagnostic effectiveness of the BRAINBox TBI Test.

The following assumptions were used to calculate sample size for the primary endpoint (within 96 h of injury) with the blood specimens collected at the first-time point: a one-sample binomial test, a clinical sensitivity of 0.85 and specificity of 0.75, a “difference to detect” of 0.07, alpha of 0.05 and a power of 0.80. The assumptions for sensitivity and specificity are based on findings from HeadSMART pilot trial (30).

The sample size is based on the binomial test for a one sample design using a two-sided significance level of α = 0.05 and a power of 1-β = 0.80 (30). The following hypothesis will be tested:


$$H_{01}:Se=Se_{0}and\;H_{02}:Sp=Sp_{0}(null\;hypotheses)$$

$$H_{\text{a1}}:Se\neq Se_{0}\;and\;H_{\text{a2}}:Sp\neq Sp_{0}(alternative\;hypothesis)$$

Where *Se* (sensitivity) is the proportion of target condition positive subjects that yield a positive test result, *Sp* (specificity) is the proportion of target condition absent subjects that yield a negative test result, Se_0_ is the desired sensitivity and Sp_0_ is the desired specificity.

Generically for both sensitivity and specificity, let p_0_ represent the null value, p_1_ the alternative and let *n* and *m* represent the sample size and the number of observed outcomes of interest, respectively. Based on the binomial distribution, the sample size may be calculated using a numerical approach by solving two equations simultaneously. For a given two-sided significance level α (Type I error), there exists a critical value *c* (nonnegative integer), such that:


$$\sum_{i=c}^{n}\left(\begin{array}{c} n\\ i \end{array}\right)p_{0}^{i}{\left(1-p_{0}\right)}^{n-i}\leq 1-\alpha /2$$

and


$$\sum_{i=c}^{n}\left(\begin{array}{c} n\\ i \end{array}\right)p_{1}^{i}{\left(1-p_{1}\right)}^{n-i}\leq 1-\beta$$

If m ≥ c, then the null hypothesis is rejected at α significance level. There exists the smallest sample size *N*, such that as long as *n* ≥ N, the power is always greater than or equal to 1-β. Then, *N* is the sample size from exact binomial testing.

### Diagnosis of ATE

Prior to algorithm development, data will be divided into training and validation sets, with a model being derived using the training data. The training phase will include examination of potentially confounding and interacting variables (e.g., age, sex, time from injury until blood draw). Any confounding variables will be included as covariates in the model. Variables with insignificant interaction, as determined by backward selection, will be omitted. The model will provide a probability/score that a subject has ATE. The c-statistic will be reported, and a threshold selected to ensure the model meets the minimum sensitivity and specificity of 85 and 75%, respectively, with confidence intervals, positive predictive value and negative predictive value reported. Accuracy will be reported as the percentage of all suspected TBI subjects correctly classified as “ATE,” vs. “no ATE,” using the identified threshold. Validation data will be derived in an independent subject set (validation cohort), against which the developed model will be tested for generalizability. *P*-values comparing the AUC, sensitivity, specificity, and accuracy will be reported, with alpha defined as < 0.05. Sample size calculation was performed in PASS statistical software (32) using an assumed prevalence of 50% ATE in the subjects enrolled with blunt head injury. The final number of evaluable subjects for the “ATE,” and “no ATE” cohorts, should each not exceed 240, which makes the total number of evaluable subjects 480. An equal number of subjects are needed for algorithm validation (*n* = 480).

### Prognosis of ATE

A separate model will be fit for symptoms at each post-enrollment time point, with data evaluated during the modeling phase, to determine the prognostic output report. Results will be grouped into low and high risk. Based on the data from the modeling/training phase, the symptom categories that are statistically supported, with sufficient prevalence to make a justified prognostic claim, will be reported. If applicable, symptoms categories may be combined into one overarching composite result, and defined as either low or high risk. Data handling for modeling will be the same as the diagnostic analysis, with the models identifying the likelihood that a subject will experience ATE symptoms at 14, 30, and 90 days.

Sample size for TBI subjects adjudicated as high or low risk for ATE symptoms at 14, 30 and 90-days post-injury is based on estimated prevalence of 60% of subjects having unresolved or emergent injury-related symptoms in the suspected ATE population at each time point (30). Using published and internal information on prevalence, as well as predefined sensitivity, specificity, confidence intervals, error, the total number of ATE subjects needed was calculated. The number of subjects with high risk of outcomes needed, the target condition for the prognosis, will be 236 evaluable subjects. The number of subjects without ATE will be 157, yielding a total number of subjects of 393 for algorithm training. With the estimated prevalence of ATE being 50%, a total of 786 blunt head trauma subjects is needed for algorithm training. This study design uses the same sample sizes for both training and validation phases, just as in the diagnostic models. Therefore, the same number of blunt head trauma subjects (*n* = 786) are needed for validation. To provide sufficient data for prognosis modeling in training and validation cohorts, up to 1,600 suspected ATE subjects will be needed. Enrollment numbers are subject to increase or decrease pending on true prevalence, results of data analysis and/or attrition.

The data collected in the clinical study that is directly related to study endpoints follows a BRAINBox Solutions blinding plan. Subjects from the training phase of the ATE diagnosis cohort may also be used in the training phase of the prognostic evaluation. Subjects from the validation phase of the ATE diagnosis cohort may be used in the validation phase of the prognostic evaluation. Therefore, the total number of suspected ATE patients should not exceed 1,600. In the event the actual prevalence differs from the assumed prevalence, the study design will include 2,000 blunt head trauma subjects as a maximum enrollment number.

## Conclusions

This protocol proposes to collect data using a multi-modality approach, including blood biomarkers, clinical characteristics, neurocognitive and neuropsychological assessments, to develop diagnostic and prognostic algorithms for ATE. The validation phase will determine the accuracy of the BRAINBox ATE Test as an aid to the diagnosis of ATE, and as an objective determination of a patient's risk to develop post-concussive symptoms.

## Ethics Statement

The studies involving human participants were reviewed and approved by the Local Ethics Committee of each participating institution. The patients/participants provided their written informed consent to participate in this study.

## Author Contributions

WP wrote the first draft. All authors contributed to the article and approved the submitted version.

## Funding

This study received funding from BRAINBox Solutions, Inc. The funder had the following involvement with the study: Study design. All authors were investigators, with their institutions receiving remuneration for the costs to perform this trial.

## Conflict of Interest

This study received funding from Brainbox, Inc. The funder had the following involvement with the study: study design. All authors were investigators, with their institutions receiving remuneration for the costs to perform this trial.

## Publisher's Note

All claims expressed in this article are solely those of the authors and do not necessarily represent those of their affiliated organizations, or those of the publisher, the editors and the reviewers. Any product that may be evaluated in this article, or claim that may be made by its manufacturer, is not guaranteed or endorsed by the publisher.
